# Self-propagating high-temperature synthesis of nano-TiC*_x _*particles with different shapes by using carbon nano-tube as C source

**DOI:** 10.1186/1556-276X-6-515

**Published:** 2011-08-31

**Authors:** Shenbao Jin, Ping Shen, Dongshuai Zhou, Qichuan Jiang

**Affiliations:** 1 Key Laboratory of Automobile Materials, Ministry of Education, People's Republic of China; 2 Department of Materials Science and Engineering, Jilin University, No. 5988 Renmin Street, Changchun 130025, People's Republic of China

**Keywords:** self-propagating high-temperature synthesis (SHS), carbon nanotubes, nano-TiC*_x _*particles

## Abstract

With using the carbon nano-tube (CNT) of high chemical activity, nano-TiC*_x _*particles with different growth shapes were synthesized through the self-propagating high temperature in the 80 wt.% metal (Cu, Al, and Fe)-Ti-CNT systems. The growth shapes of the TiC*_x _*particles are mainly octahedron in the Cu- and Al-Ti-CNT systems, while mainly cube- and sphere-like in the Fe-Ti-CNT system.

## Introduction

As known, some ceramic particles, such as titanium carbide (TiC*_x_*), are usually used as the reinforcing phases in the composites due to their unique properties such as high melting point, extreme hardness, and high resistance to corrosion and oxidation. Recently, many experimental and theoretical studies have indicated that decreasing the sizes of the reinforcing ceramic particulates can lead to substantial improvements in mechanical performance of the composites [1-11]. For example, Ma et al. [11] showed that the tensile strength of 1 vol.% Si_3_N_4 _(10 nm)/Al composite is comparable to that of the 15 vol.% SiC_p _(3.5 μm)/Al composite, and the yield strength of the former is much higher than that of the latter. Then, with significantly increasing intention to develop nanoparticle-reinforced composites with superior mechanical properties, the demand for nano-sized ceramic powders, including TiC*_x_*, has become more urgent.

Among the variety of the preparation methods for TiC*_x_*, self-propagating high-temperature synthesis (SHS) is noted by us because it is a convenient and efficient way to synthesize TiC*_x_*. However, the SHS is quite challenging to produce the nano-sized ceramic particles because the combustion temperature will lead to considerable coarsening of the ceramic particles. At present, the usual method for synthesizing the nano-ceramic particles through the SHS is the addition of volatile diluents such as NaCl into the reactants. Some nano-ceramic particles such as TiB_2 _and ZrB_2 _have been prepared by adding NaCl to the SHS reactants [12-14], and the nano-TiC*_x _*particles (20 to 100 nm) were also obtained by Nersisyan et al. [15] in the 30 wt.% NaCl-Ti-carbon black system.

On the other hand, the addition of a second metal (Me) such as Al, Cu, and Fe can also decrease the combustion temperature and thus prevent the ceramic particles from further growth. For example, with the increase in the Al incorporation from 10 to 40 wt.%, the sizes of the TiC*_x _*particles decrease from about 3 μm to 400 nm [16]. However, when more Me (≥50 wt.%) is incorporated, the SHS reaction tends to be incomplete or even cannot be ignited. Generally, this situation can be improved through using finer C-source particles because they can enlarge the area of the contact surface between the liquid and the carbon source and decrease the activation energy of the SHS reaction. At present, the source of C that are mostly used during the SHS are graphite (typically 1 to 150 μm) and C black (< 100 nm). In contrast to them, carbon nano-tube (CNT) has much finer size, usually 5 to 20 nm in diameter. In fact, CNT has been used to synthesize the nanostructured TiC-TiB_2 _[17] and carbide nanofibers [18] during the SHS.

In this paper, taking advantage of high chemical activity of the CNT, we tried to prepare the nano-sized TiC*_x _*particles during the SHS in the Me (Cu, Al, and Fe)-Ti-CNT systems with the high contents of the Me incorporation. The morphologies of the TiC*_x _*particles formed in these systems were investigated, and the mechanism for the difference in their morphology was discussed.

## Experimental methods

The raw materials utilized were multi-walled carbon nanotubes (20 to 30 nm in diameter and approximately 30 μm in length, purity > 95 wt.%, Chengdu Organic Chemicals Co. Ltd., Chinese Academy of Sciences, Chengdu, China), Ti powders (> 99.5% purity, approximately 48 μm, Institute of Nonferrous Metals, Beijing, China), Al powders (> 99.0% purity, approximately 48 μm, Northeast Light Alloy Ltd. Co., Harbin, China), Cu powders (> 99.5% purity, approximately 48 μm, Institute of Nonferrous Metals, Beijing, China) and Fe powders (> 99.5% purity, approximately 48 μm, Institute of Nonferrous Metals, Beijing, China). The Ti and CNT powders with a molar ratio of 1:1 were mixed with the Me (Cu, Al, and Fe) powders in relative quantities of 50, 60, 70, and 80 wt.%, respectively. The reactants were mixed sufficiently by ball milling at a low speed (approximately 35 rpm) for 6 h and then pressed into the cylindrical compacts of approximately 22 mm in diameter and approximately 15 mm in height with green densities of approximately 60 ± 2% of theoretical. The SHS experiments were conducted in a self-made vacuum vessel in an Ar atmosphere using an arc as ignition source. During the SHS process, the temperature in the position about 3 mm beneath the center of the compact top surface was measured by W5-Re26 thermocouples, and the signals were recorded and processed by a data acquisition system using an acquisition speed of 50 ms per point.

The phase compositions in the reacted samples were identified by X-ray diffraction (XRD, Rigaku D/Max 2500PC, Rigaku Corporation, Tokyo, Japan) with CuKα radiation using a scanning speed of 4°/min. The reacted Cu-Ti-CNT samples were then dissolved in a saturated FeCl_3 _water solution, and the reacted Al- and Fe-Ti-CNT samples were dissolved in an 18 vol.% HCl-distilled water solution, to remove the Me coatings on the surfaces of the TiC*_x _*particles. The morphologies of the extracted TiC*_x _*particles were observed using a field emission scanning electron microscope (FESEM, JSM 6700F, JEOL, Tokyo, Japan) and a transmission electron microscope (TEM, JSM 200EX, JEOL).

## Results and discussion

**Figure 1:**
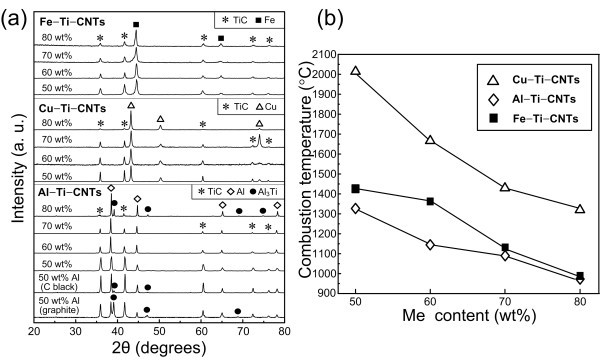
**XRD patterns of SHS products and the variation in the maximum combustion temperature**. (**a**) XRD patterns of the SHS products and (**b**) the variation in the maximum combustion temperature with the Me content.

In the Me-Ti-C systems, the Me-Ti liquid forms firstly during the heating. The carbon then diffuses into the Me-Ti liquid, and when a critical concentration is achieved, the TiC*_x _*begins to form by reaction between [C] and [Ti]. Accordingly, the diffusion of carbon in the molten metals is a key step to form TiC*_x_*, and thus different carbon sources, i.e., graphite and C black, have great effects on the product morphology and the reaction rate of [Ti] and [C] to form TiC*_x_*. Generally speaking, the carbon source with finer sizes will make the combustion reaction proceed more thoroughly. For example, when C black was used as the carbon source in 50 wt.% Al-Ti-C system, the content of the intermediate phase Al_3_Ti decreases greatly than that of the graphite being used as the carbon source (Figure 1a). In contrast to the graphite and C black, carbon nano-tube (CNT) has much finer sizes. Furthermore, the defects such as pentagons, heptagons and vacancies in the structure of the CNT endow it with more chemical activity [19,20]. Therefore, the CNT will dissolve more rapidly in the liquid Me to provide dissociated [C], which promotes the SHS reaction. This speculation was proved as there is no Al_3_Ti formed in the 50 wt.% Al-Ti-CNT system. Actually, only when the Al content was increased to 80 wt.% in the Al-Ti-CNT system, a little amount of Al_3_Ti formed. In Cu- and Fe-Ti-CNT systems, within the range of 50 to 80 wt.% for the Me content, no Al_3_Ti is formed.

As known, according to Merzhanov's empirical criterion, for the reaction to be self-sustaining in the absence of preheat, the adiabatic temperature (*T*_ad_) should not be less than 1,800 K, corresponding to the maximum addition of 67.12 wt.% Cu, 46.65 wt.% Al [16], and 77.4 wt.% Fe [21] in the Me-Ti-C systems, respectively. However, in our experiments, because of the high activity of the CNT, the samples with 70 wt.% Al and 80 wt.% Cu and Fe can be ignited easily. Figure 1b shows the variation in the maximum combustion temperature with the Me content. Clearly, the maximum combustion temperature in all the systems decreases as the Me content increases, and the sequence is *T*_Cu-Ti-CNT _>*T*_Fe-Ti-CNT _>*T*_Al-Ti-CNT_. The difference in the combustion temperature in these systems, of course, will have an important influence on the shape and size of the synthesized TiC*_x _*particles.

**Figure 2:**
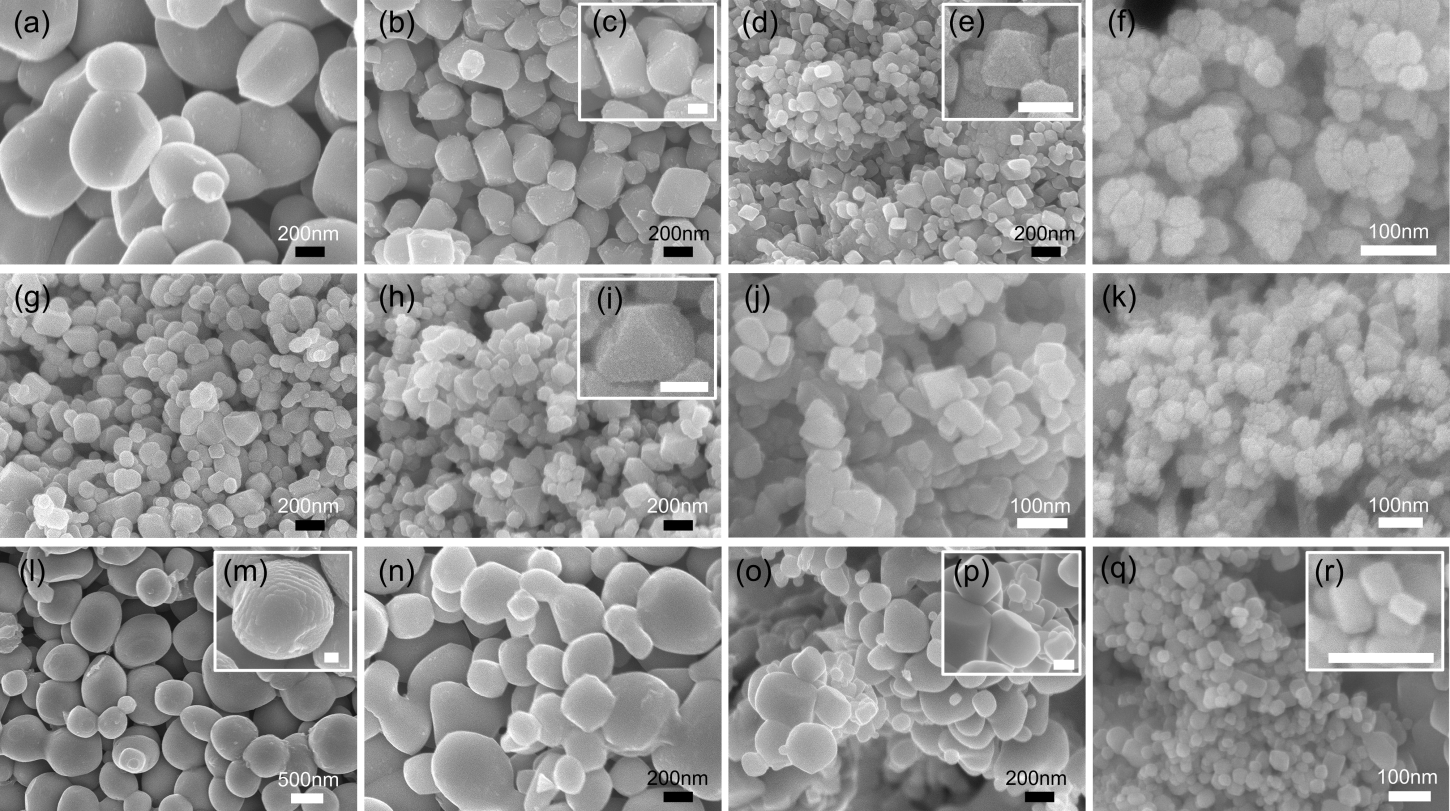
**Morphologies of the TiC*_x _*particles formed in the Me-Ti-CNT systems**. (**a**) 50 wt.% Cu, (**b**, **c**) 60 wt.% Cu, (**d**, **e**) 70 wt.% Cu, (**f**) 80 wt.% Cu, (**g**) 50 wt.% Al, (**h**, **i**) 60 wt.% Al, (**j**) 70 wt.% Al, (**k**) 80 wt.% Al, (**l**, **m**) 50 wt.% Fe, (**n**) 60 wt.% Fe, (**o**, **p**) 70 wt.% Fe, and (**q**, **r**) 80 wt.% Fe. The scale bars in the inset images represent 100 nm.

As indicated in Figure 2, with increasing the Me content, the TiC*_x _*particles formed in the Cu-, Al-, and Fe-Ti-CNT systems show a significant decrease in size. In the sample with 50 wt.% Cu, the sizes of the TiC*_x _*particles are about 600 nm (Figure 2a), while when the Cu content increases to 60, 70, and 80 wt.%, the sizes of the TiC*_x _*particles decrease to about 400, 100, and 60 nm, respectively (Figure 2b, d, f). Accompanying the decrease in the particle size, the TiC*_x _*particles change their shapes from sphere-like to regular octahedron (Figure 2c, e). The same growth shape as octahedron can be also observed in the TiC*_x _*particles formed in the samples with 50, 60, and 70 wt.% Al (Figure 2g, h, j), of which the particle sizes are about 200, 150, and 70 nm, respectively. When the Al content is increased to 80 wt.%, the shape of the TiC*_x _*particles cannot be observed clearly, and the particle size decreases to about 40 nm (Figure 2k). As we have suggested before, in the Al-Ti-C system, the TiC*_x _*particles grow through the deposition and lateral stacking of the growth units on the (111) surfaces [22,23]. In contrast to the growth mode of the TiC*_x _*particles in the Al-Ti-CNT system, the TiC*_x _*particles growing in the Fe-Ti-CNT system have a different growth mode, i.e., the lateral stack along the (100) surfaces (Figure 2m). Under this mode, the TiC*_x _*particles should grow into the cubic shapes. However, because of the round turning of the (100) surfaces, most of the TiC*_x _*particles in the Fe-Ti-CNT system show the sphere-like shapes (Figure 2l). When the Fe content increases, the sizes of the TiC*_x _*particles decrease and the cubic character of the TiC*_x _*particles becomes more and more distinct (Figure 2h). In the sample with 70 wt.% Fe, there are many TiC*_x _*particles with regular cubic shapes and sizes of about 200 nm (Figure 2o). Increasing the Fe content to 80 wt.% further decreases the sizes of the TiC*_x _*particles to approximately 70 nm, with primarily cubic shapes (Figure 2q).

**Figure 3:**
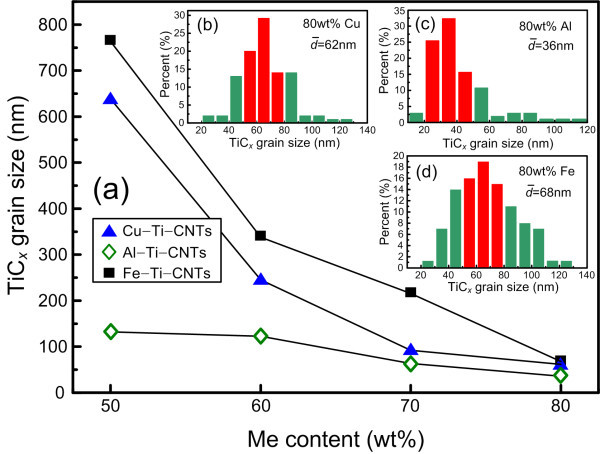
**Mean sizes and the size distribution of the TiC*_x _*particles**. (**a**) Mean sizes calculated based on the statistic analysis of a hundred of TiC*_x _*particles in the FESEM images. (**b**, **c**, **d**) Size distribution of the TiC*_x _*particles formed in the samples with 80 wt.% Cu, Al, and Fe, respectively.

**Figure 4:**
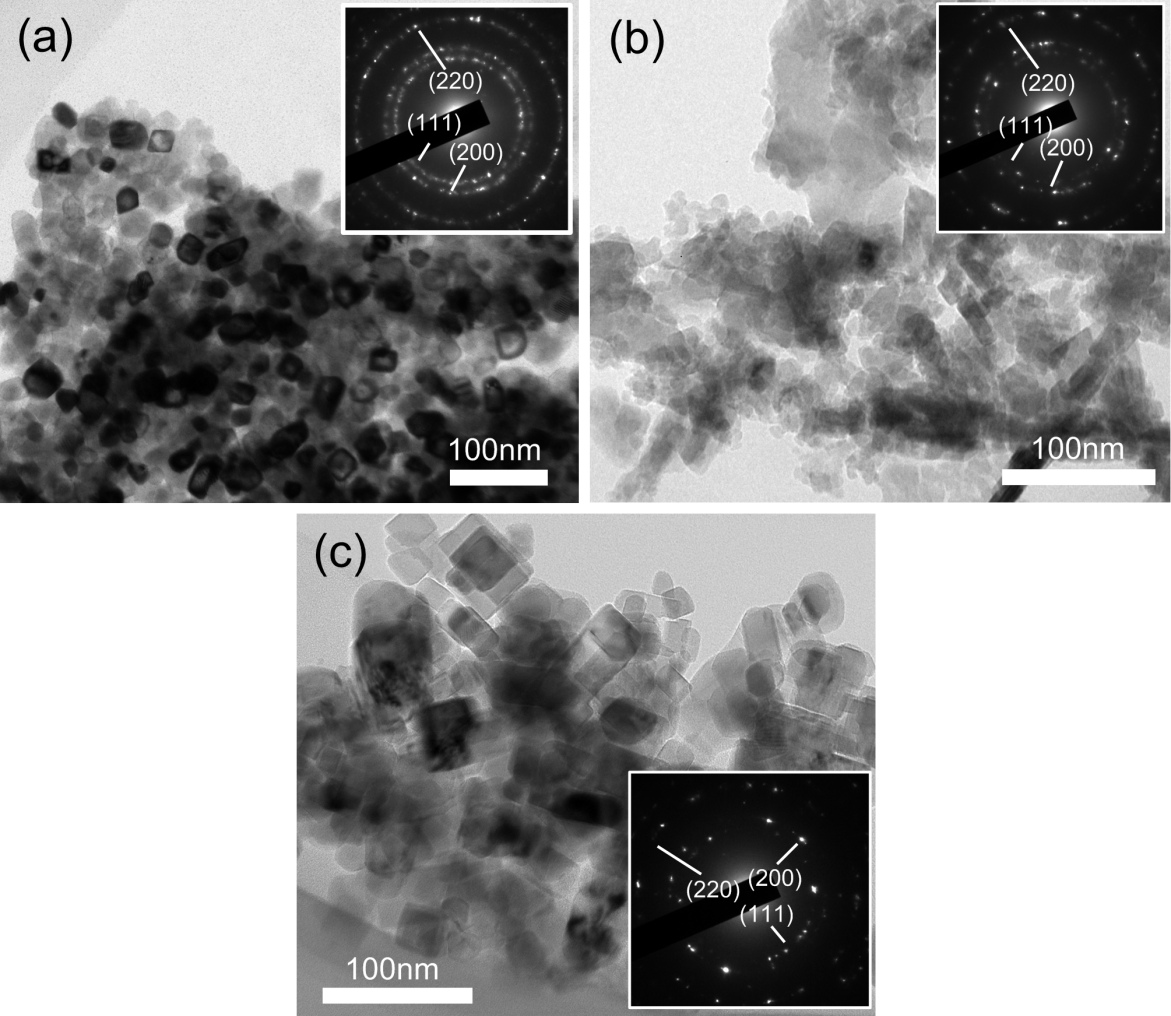
**TEM images of the TiC*_x _*particles formed in the Me-Ti-CNT samples**. (**a**) 80 wt.% Cu, (**b**) 80 wt.% Al, and (**c**) 80 wt.% Fe. Inset images show the corresponding diffraction rings.

Figure 3a gives the mean sizes based on the statistic analysis of a hundred of TiC*_x _*particles in the FESEM images for the Me-Ti-CNT systems. The decrease in the TiC*_x _*particle sizes with the increase in the Me content is easy to understand because of the decreasing combustion temperature. When the Me content increases to 80 wt.% for Cu, Al, and Fe, the sizes of the TiC*_x _*particles decrease to about $62_{-38}^{+60}$, $36_{-20}^{+80}$, and $68_{-40}^{+58}$ nm, respectively. Furthermore, it can be noticed that in the above Me-Ti-CNT systems, the TiC*_x _*particles formed in the Al-Ti-CNT samples are the finest, which could be attributed to the lowest combustion temperatures. Nevertheless, the TiC*_x _*particles formed in the Fe-Ti-CNT samples have the largest sizes even though their combustion temperatures are quite lower than those formed in the Cu-Ti-CNT samples. This phenomenon is meaningful to the discussion in the following paragraphs on the mechanism of the TiC*_x _*shape variation with the different kinds of the Me addition. Figure 4 gives the TEM images of the TiC*_x _*particles formed in the samples with 80 wt.% Me. The diffraction rings from inner to outer in the inserted images in Figure 4a, b, c match the (111), (200), and (220) planes of the fcc TiC.

As we have mentioned, the shapes of the TiC*_x _*particles vary considerably in the different kinds of the Me incorporated Ti-CNT systems, i.e., the TiC*_x _*particles formed in the Cu- and Al-Ti-CNT systems are mainly with the octahedral shapes, while those formed in the Fe-Ti-CNT system are mainly with the cubic and sphere-like shapes. In our pervious paper [23], we have suggested that the growth shapes of the TiC*_x _*particles in the Al-Ti-C system should be directly related to their stoichiometry (*x*), i.e., when the stoichiometry is low, the TiC*_x _*(111) surfaces are the most stable and the growth shape is octahedron, while when the stoichiometry increases, the free energy of the (111) surfaces increases, which leads to the diminishing in the (111) surfaces on the TiC*_x _*crystals and the exposure of the (100) surfaces. According to this speculation, the stoichiometry of the TiC*_x _*crystals formed in the Cu- and Al-Ti-CNT systems should be low and that in the Fe-Ti-CNT system should be high. Here, we qualitatively estimate the stoichiometry of the TiC*_x _*formed in the combustion stage based on the phenomenon that the TiC*_x _*particles grown in the Fe-Ti-CNT samples are the largest while their combustion temperatures are relatively low. As known, carbon has good chemical affinity with Fe. Hence, the carbon atoms could dissolve rapidly in the Fe melt, which leads to the formation of the C-rich regions near the CNTs at the initial stage of the SHS. In these C-rich regions, the TiC*_x _*particles form and grow rapidly. That is why the sizes of the TiC*_x _*particles formed in the Fe-Ti-CNT system are generally large even though their combustion temperatures are quite low. As another consequence of the high C concentration, the stoichiometry of these primitively formed TiC*_x _*particles in the Fe melt is relatively high. Then, the (100) surfaces of TiC*_x _*are stable and the growth shape is cube. For the Cu- and Al-Ti-CNT systems, the CNT dissolves more slowly because of the poor chemical reactivity between carbon and the Cu (or Al) melt as well as very limited solubility of carbon in molten Cu and Al. In this case, the TiC*_x _*forms and grows under a condition of C scarcity. Hence, the TiC*_x _*particles grown in these two melts are with relatively small sizes, and the TiC*_x _*stoichiometry formed at the combustion stage is low. Accordingly, the TiC*_x _*growth shape is octahedron.

Frankly speaking, spending a great amount of metal (Al/Cu/Fe) to only synthesize the TiC*_x _*nanoparticles is really uneconomical. Nevertheless, considering that the TiC*_x _*particles reinforced metal matrix composites can be fabricated conveniently through following a pressing or forging treatment after the SHS [24], the real significance of this research is to provide a perspective to *in situ *synthesize the nano-TiC*_x _*particle reinforced composites more conveniently by using CNT. As known, the fabrication of ceramic nanoparticles reinforced metal matrix is an important development direction for the development of composites, and many papers have been published on this issue from 2000. In 99% of these works, the nanoparticles were introduced into the metal matrix through external addition. In these methods, the mixing of nano-sized particles in metal liquid is usually lengthy, expensive, and energy consuming. In fact, in contrast with the external addition methods, the method with nanoparticles *in situ *synthesis has the advantages of a more homogeneous distribution of the nanoparticles, clearer interface between nanoparticles and matrix, and lower chances to introduce impurity. However, when metal matrix is with high content (≥50 wt.%), the TiC*_x _*formation reaction tends to be incomplete or even cannot be ignited by using traditional C sources such as C black or graphite. To solve this key question in the SHS, we used CNT as the C source in this paper. The results indicate that the samples with more than 70 wt.% metals can still be ignited easily because of the high activity of the CNT. In fact, in our following study, by using CNT as C source, we have successfully *in situ *synthesized the TiC*_x _*nanoparticles in 97 wt.% Cu matrix, and the composite was fabricated conveniently by the SHS and a subsequent pressing or forging process. Moreover, our results suggest that other nano-sized transition metal carbides (such as SiC, ZrC, and NbC) and the corresponding reinforced composites could also be synthesized with using the high chemical activity of the CNT.

## Conclusions

The using of CNT increases the reactivity in the Me (Cu, Al, and Fe)-Ti-CNT systems and makes SHS reaction more easily ignited. The sizes of the synthesized TiC*_x _*particles decrease with the increase in the Me content. When the Me content increases to 80 wt.% for Cu, Al, and Fe, the sizes of the TiC*_x _*particles decrease to about $62_{-38}^{+60}$, $36_{-20}^{+80}$, and $68_{-40}^{+58}$ nm, respectively. The shapes of the nano-TiC*_x _*particles formed in the Cu- and Al-Ti-CNT systems are mainly octahedral, while those formed in the Fe-Ti-CNT system are mainly cubic and sphere-like. This shape variation of the TiC*_x _*formed in different kinds of the Me liquid environment is believed to relate to the different stoichiometries of the TiC*_x _*formed during the combustion stage in these systems.

## Competing interests

The authors declare that they have no competing interests.

## Authors' contributions

All the authors contributed to writing of the manuscript. SBJ carried out the experiments under the instruction of QCJ.

## References

[B1] Kang YC Chan SL Tensile properties of nanometric Al_2_O_3 _particulate-reinforced aluminum matrix composites Mater Chem Phys 2004 85 438 443 10.1016/j.matchemphys.2004.02.002

[B2] Liu YQ Cong HT Wang W Sun CH Cheng HM AlN nanoparticle-reinforced nanocrystalline Al matrix composites: fabrication and mechanical properties Mater Sci Eng A 2009 505 151 156 10.1016/j.msea.2008.12.045

[B3] Hesabi ZR Hafizpour HR Simchi A An investigation on the compressibility of aluminum/nano-alumina composite powder prepared by blending and mechanical milling Mater Sci Eng A 2007 454-455 89 98

[B4] Hemanth J Development and property evaluation of aluminum alloy reinforced with nano-ZrO_2 _metal matrix composites (NMMCs) Mater Sci Eng A 2009 507 110 113 10.1016/j.msea.2008.11.039

[B5] Woo KD Zhang DL Fabrication of Al-7wt%Si-0.4wt%Mg/SiC nanocomposite powders and bulk nanocomposites by high energy ball milling and powder metallurgy Curr Appl Phys 2004 4 175 178 10.1016/j.cap.2003.11.002

[B6] Ying DY Zhang DL Processing of Cu-Al_2_O_3 _metal matrix nanocomposite materials by using high energy ball milling Mater Sci Eng A 2000 286 152 156 10.1016/S0921-5093(00)00627-4

[B7] Hassan SF Gupta M Development of high performance magnesium nano-composites using nano-Al_2_O_3 _as reinforcement Mater Sci Eng A 2005 392 163 168 10.1016/j.msea.2004.09.047

[B8] Lee CJ Huang JC Hsieh PJ Mg based nano-composites fabricated by friction stir processing Scr Mater 2006 54 1415 1420 10.1016/j.scriptamat.2005.11.056

[B9] Wong WLE Gupta M Improving overall mechanical performance of magnesium using nano-alumina reinforcement and energy efficient microwave assisted processing route Adv Eng Mater 2007 9 902 909 10.1002/adem.200700169

[B10] Artzt E Size effects in materials due to microstructural and dimensional constraints: a comparative review Acta Mater 1998 46 5611 5626 10.1016/S1359-6454(98)00231-6

[B11] Ma ZY Tjong SC Li YL Liang Y High temperature creep behavior of nanometric Si3N4 particulate reinforced aluminium composite Mater Sci Eng A 1997 225 125 134 10.1016/S0921-5093(96)10870-4

[B12] Khanra AK Pathak LC Mishra SK Godkhindi MM Effect of NaCl on the synthesis of TiB_2 _powder by a self-propagating high-temperature synthesis technique Mater Lett 2004 58 733 738 10.1016/j.matlet.2003.06.003

[B13] Khanra AK Pathak LC Mishra SK Godkhindi MM Self-propagating-high-temperature synthesis (SHS) of ultrafine ZrB_2 _powder J Mater Sci Lett 2003 22 1189 1191 10.1023/A:1025336230885

[B14] Camurlu HE Maglia F Preparation of nano-size ZrB_2 _powder by self-propagating high-temperature synthesis J Eur Ceram Soc 2009 29 1501 1506 10.1016/j.jeurceramsoc.2008.09.006

[B15] Nersisyan HH Lee JH Won CW Self-propagating high-temperature synthesis of nano-sized titanium carbide powder J Mater Res 2002 17 2859 2864 10.1557/JMR.2002.0415

[B16] Song MS Huang B Zhang MX Li JG Study of formation behavior of TiC ceramic obtained by self-propagating high-temperature synthesis from Al-Ti-C elemental powders Int J Refractory Met Hard Mater 2009 27 584 589 10.1016/j.ijrmhm.2008.09.009

[B17] Deorsola FA Atias Adrian IC Ortigoza Villalba GA DeBenedetti B Nanostructured TiC-TiB_2 _composites obtained by adding carbon nanotubes into the self-propagating high-temperature synthesis process Mater Res Bull 2011 46 995 999 10.1016/j.materresbull.2011.03.017

[B18] Lia XK Westwood A Brown A Brydson R Rand B A convenient, general synthesis of carbide nanofibres via templated reactions on carbon nanotubes in molten salt media Carbon 2009 47 201 208 10.1016/j.carbon.2008.09.050

[B19] Charlier JC Defects in carbon nanotubes Acc Chem Res 2002 35 1063 1069 10.1021/ar010166k 12484794

[B20] Mintmire JW White CT Electronic and structural properties of carbon nanotubes Carbon 1995 33 893 902 10.1016/0008-6223(95)00018-9

[B21] Saidi A Chrysanthou A Wood JV Kellie JLF Characteristics of the combustion synthesis of TiC and Fe-TiC composites J Mater Sci 1994 29 4993 4998 10.1007/BF01151089

[B22] Jin SB Shen P Zou BL Jiang QC Morphology evolution of TiC*_x _*grains during SHS in an Al-Ti-C system Cryst Growth Des 2009 9 646 649 10.1021/cg800527q

[B23] Jin SB Shen P Lin QL Zhan L Jiang QC Growth mechanism of TiC*_x _*during self-propagating high-temperature synthesis in an Al-Ti-C system Cryst Growth Des 2010 10 1590 1597 10.1021/cg9010983

[B24] Shu SL Lu JB Qiu F Xuan QQ Jiang QC Effects of alloy elements (Mg, Zn, Sn) on the microstructures and compression properties of high-volume-fraction TiC*_x_*/Al composites Scr Mater 2010 63 1209 1211 10.1016/j.scriptamat.2010.08.040

